# Selected Vascular, Inflammatory, and Lipid Parameters in Patients with Selected Keratinization Disorders: Preliminary Data from a Retrospective Observational Study

**DOI:** 10.3390/jcm15135179

**Published:** 2026-07-02

**Authors:** Aldona Pietrzak, Jakub Kęsik, Radosław Mlak, Katarzyna Wertheim-Tysarowska, Dariusz Matosiuk, Bartłomiej Wawrzycki

**Affiliations:** 1 Department of Dermatology, Venereology and Paediatric Dermatology, Medical University of Lublin, 20-080 Lublin, Poland; 2 Department of Vascular Surgery and Angiology, Medical University of Lublin, 20-081 Lublin, Poland; 3 Department of Laboratory Diagnostics, Medical University of Lublin, 20-059 Lublin, Poland; 4 Department of Medical Genetics, Institute of Mother and Child, 01-211 Warsaw, Poland; 5 Department of Synthesis and Chemical Technology of Pharmaceutical Substances, Faculty of Pharmacy, Medical University of Lublin, 20-059 Lublin, Poland

**Keywords:** keratinization disorders, genodermatoses, systemic inflammation, lipid metabolism, interleukin-17A, intima-media thickness

## Abstract

**Background:** Recent research has drawn attention to potential systemic inflammation in patients with inherited epidermal differentiation disorders (EDD)—rare genetic skin conditions that may contribute to vascular dysfunction. **Objective:** This study assessed selected vascular, inflammatory, and biochemical parameters in 20 patients with selected EDD and 20 matched healthy controls. **Methods:** Assessments included Doppler ultrasound, ankle-brachial index, intima-media thickness (IMT), and laboratory profiling focused on lipid metabolism and systemic inflammation. **Results:** EDD patients showed significantly lower LDL cholesterol and higher interleukin-17A (IL-17A) than controls. IMT values were similar across groups and disease types, but correlated positively with age, body weight, waist circumference, triglycerides, and glucose, and negatively with reactive lymphocytes. No link was found between IMT and self-reported cardiovascular symptoms. **Conclusions:** In patients with selected EDD, we observed a distinct biochemical profile characterized by lower LDL cholesterol and higher IL-17A concentrations, without accompanying structural arterial changes—carotid IMT and ABI did not differ from controls and remained stable at short-term follow-up. These alterations may reflect disease-specific disturbances of lipid and inflammatory homeostasis rather than classical atherosclerosis. Although they could be of theoretical relevance to long-term vascular health, no structural arterial abnormality was demonstrated. Longitudinal studies incorporating endothelial function testing are needed to clarify their significance.

## 1. Introduction

Recent studies have indicated the complex systemic interactions between dermatological conditions and cardiovascular diseases (CVD) owing to shared pathogenic pathways involving inflammation, oxidative stress, immune dysregulation and, often, lipid abnormalities [1,2,3,4,5]. An example is psoriasis [6], where proinflammatory cytokines, e.g., interleukin-17 (IL-17), IL-23, tumor necrosis factor-alpha, and C-reactive protein (CRP) are frequently elevated. Recent studies have suggested that these cytokines can accelerate endothelial damage and atherogenesis [7,8,9,10]. To date, there have been no studies pointing to the involvement of these factors in genodermatoses.

Interestingly, in recent years, several studies have indicated that immunological dysregulation is significantly associated with the pathogenesis of several monogenic genodermatoses, including epidermal differentiation disorders (EDDs) like ichthyosis or epidermolysis bullosa [11,12]. Moreover, certain genodermatoses are often accompanied by dysregulated lipid metabolism and increased oxidative stress markers, factors closely associated with endothelial dysfunction and arterial plaque formation [13,14]. Also, the time-consuming and exhausting nature of the treatment may affect intima-media thickness (IMT), which may have atherothrombotic consequences [15].

Despite substantial gaps in knowledge regarding the extracutaneous manifestations of non-major ichthyoses, we hypothesized that inflammatory pathways in these disorders may extend beyond cutaneous symptoms, similarly to what has been observed in psoriasis. The aim of this study was to assess selected vascular and biochemical parameters associated with cardiovascular risk in patients with EDDs and to compare them with those of matched healthy controls.

## 2. Materials and Methods

### 2.1. Study Design

The study group included 20 patients with EDD treated between April 2019 and September 2023 at the Dermatology Outpatient Clinic and Dermatology Clinic at University Clinical Hospital No. 1 (USK-1), Lublin. The control group, initially recruited from 40 volunteers, was matched to the study group using propensity score matching (PSM) for gender (exact match) and age (allowed difference of 1 year). After PSM, both groups were equal in size (*n* = 20 each).

Information on current and recent (within the preceding two months) topical and systemic therapy, including retinoids (e.g., acitretin), was recorded for all participants.

The study included patients with a clinical diagnosis of inherited epidermal differentiation disorder (EDD), provided that their age allowed reliable clinical, vascular, and laboratory assessment. Eligible participants were required to have complete clinical records, vascular examination data, and laboratory results available for analysis. Written informed consent was obtained from all participants or from their legal guardians when applicable. The control group included age- and sex-matched healthy individuals without known inherited skin disease.

Exclusion criteria were acute infectious disease at the time of evaluation, known cardiovascular disease, previous thromboembolic event, chronic systemic inflammatory or autoimmune disease unrelated to EDD, current treatment with systemic agents known to affect lipid metabolism or inflammatory markers, and incomplete medical records or missing laboratory or ultrasound data. Control participants were additionally required to have no history or clinical evidence of inherited skin disease.

Genetic tests were performed either in the MedGen Medical Centre in Warsaw or in the Institute of Mother and Child in Warsaw.

All participants provided informed consent to participate in the study. The protocol was approved by the Bioethics Committee of the Medical University of Lublin (approval no. KE-0254/62/2017).

### 2.2. Hematological and Biochemical Tests

Venous blood samples were collected from all participants under standard conditions and analyzed in the certified laboratory of the Medical University of Lublin. Laboratory assessment included biomarkers related to lipid metabolism, systemic inflammation, liver function, mineral metabolism, and selected cardiovascular risk parameters. In particular, the evaluated variables comprised lipid profile components, glucose, inflammatory cytokines, acute-phase markers, cardiac biomarkers, selected hematological activation markers, and routine biochemical parameters. Immunological and biochemical measurements were performed using the Cobas E601 Immunology Analyzer (Roche, Indianapolis, IN, USA), the ARCHITECT i2000SR immunoassay analyzer (Abbott Laboratories, Abbott Park, IL, USA), and the Multiskan FC microplate spectrophotometer (Thermo Fisher Scientific, Waltham, MA, USA) with an automatic microplate washer, in accordance with the manufacturers’ instructions.

### 2.3. Angiologic Evaluation

All Doppler ultrasound examinations were performed at the Department of Vascular Surgery and Angiology in Lublin using a Canon Aplio A ultrasound system (Canon Medical Systems, Otawara, Tochigi, Japan) equipped with an 11L3 linear probe. A linear probe was used for the assessment of carotid arteries as well as superficial and deep veins. In obese patients, when the depth of the examined deep veins exceeded the penetration range of the linear probe, a convex probe (8C1) was used as an adjunct. Each examination was performed by one doctor. This physician was an experienced specialist in vascular surgery certified by the Ultrasound Society.

Initially, the carotid arteries were visualized in the transverse plane and subsequently in the longitudinal plane, with optimal depth settings and focal zone adjusted to the level of the examined vessel. IMT measurements were performed using high-resolution superficial imaging. IMT was assessed bilaterally in the common carotid arteries, 1–2 cm proximal to the bifurcation, with all measurements obtained from the far wall. For each artery, IMT was measured at five predefined points, and the mean value was used for analysis.

The assessment of the venous system in the lower limbs was performed using the same ultrasound system with linear and, when necessary, convex probes. The screening for venous insufficiency focused on the lower extremities, as involvement of the upper extremities represents a negligible proportion of chronic venous disease, largely due to lower hydrostatic pressure in upper limb veins compared to the lower limbs. Morphological evaluation included examination of all venous segments from the inferior vena cava to the ankles, with the aim of assessing vein patency and identifying thrombi or post-thrombotic changes such as residual obstruction or fibrosis [16,17].

Ankle-Brachial Index (ABI) was measured in the supine position using a sphygmomanometer Microlife Gentle (Microlife AG, Widnau, Switzerland) and a continuous wave Doppler instrument (Canon Otawara, Japan). Systolic pressure was determined bilaterally on the brachial arteries (the higher value was used for further calculations) and then bilaterally on the posterior tibial and dorsal foot arteries. The systolic quotient was calculated.

Valve closure timing was measured in a standing position, with the assessment of deep veins at the knee level and superficial veins (including the great saphenous and small saphenous veins).

In the study group, IMT and ABI were repeated after 3 months to assess short-term intra-individual variability and measurement reproducibility in this rare-disease cohort. This interval is too short to capture structural atherosclerotic progression; the repeat assessment was therefore intended primarily as a stability and quality-control check rather than as a measure of disease progression.

### 2.4. Statistical Analysis

MedCalc 15.8 software (MedCalc Software Ltd., Ostend, Belgium) was used for statistical analysis. The Fisher’s exact test or the chi-square test was applied to assess differences between categorized variables with two or more than two groups of variants, respectively. The D’Agostino–Pearson test was used to assess the distribution of continuous data. As the data distribution differed from normal, nonparametric tests were employed for further analysis. Data clustering was reported as the median and dispersion with the minimum–maximum (min–max) range and the interquartile range (IQR). Differences in continuous variables between the groups were analyzed using the Mann–Whitney U test for independent variables or the Wilcoxon test for paired variables. The Spearman rank correlation test was used to assess correlations between selected continuous variables. Given the exploratory nature of the study and the relatively small sample size, no correction for multiple testing was applied; accordingly, all reported associations should be regarded as hypothesis-generating. Statistical significance was defined at *p* < 0.05.

## 3. Results

### 3.1. Baseline Characteristics

The patient cohort included:

- Twelve patients with generalized skin lesions (nonsyndromic-nEDD or syndromic-sEDD): ALOX12B-nEDD (*n* = 4); TGM1-nEDD (*n* = 2); and single cases of KRT10-nEDD; FLG-nEDD; CYP4F22-nEDD; MBTPS2-sEDD; JUP-sEDD; SPINK5-sEDD;
- Six patients with palmoplantar keratoderma (pEDD): KRT1-pEDD (*n* = 2); KRT9-pEDD (*n* = 2); DSG1-pEDD (*n* = 1), and AAGAB-pEDD (*n* = 1).
- Two patients without molecular analysis: erythrokeratodermia variabilis-like (*n* = 1), palmoplantar keratoderma (*n* = 1).

Baseline characteristics of the study group are presented in Table 1.

### 3.2. Comparison of the Demographic Data, Cardiovascular Risk Factors, and Clinical and Laboratory Parameters in the Study and Control Groups

No significant differences in weight, blood pressure, lipid profile (except for low-density lipoprotein [LDL] cholesterol levels), glucose, and NT-proBNP were observed between groups (Table 2).

Significantly lower LDL cholesterol levels were found in the study group compared to controls (*p* = 0. 0262).

**Figure 1:**
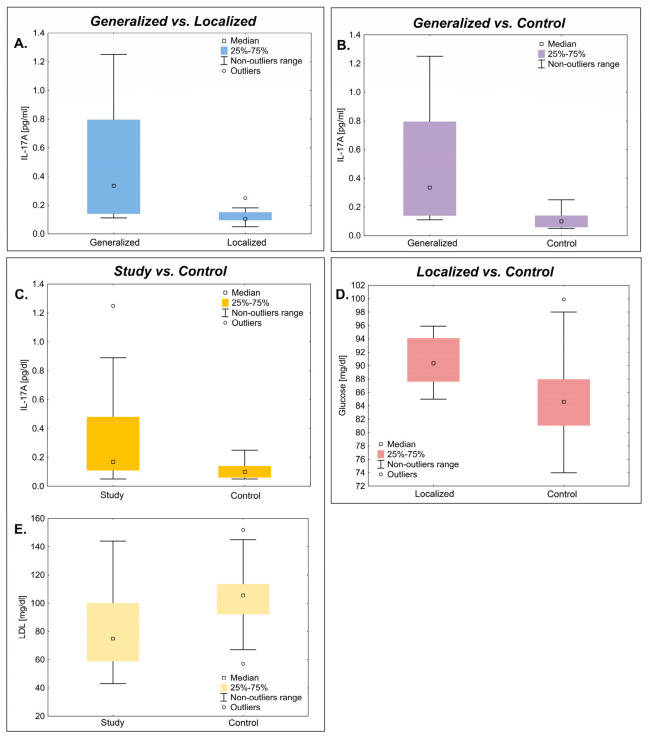
A graphical presentation of selected statistically significant differences observed between the groups analyzed. The box plots show the concentrations of IL-17A (**A**–**C**), glucose (**D**) and LDL cholesterol (**E**) according to the study groups and in comparison with the control group. The line inside the box represents the median, the box encompasses the interquartile range (IQR), and the whiskers represent the range of non-outlier values. The dots indicate outliers.

IL-6, CRP, and the remaining biochemical parameters did not differ significantly between the study and control groups; the only significant inflammatory difference was a higher IL-17A concentration in the study group (*p* = 0.0047) and, on subgroup analysis, in patients with generalized disease (*p* = 0.0002 vs. controls) (Table 2, Figure 1).

### 3.3. Comparison of Mean Intima-Media Thickness and Ankle-Brachial Index Results

There were no significant differences in the mean IMT and ABI measurements between the study group (including subgroups with generalized and localized disease) and the control group (Table 3).

No significant changes in IMT or ABI were observed between baseline and the 3-month follow-up in the study group (Table 4).

**Figure 2:**
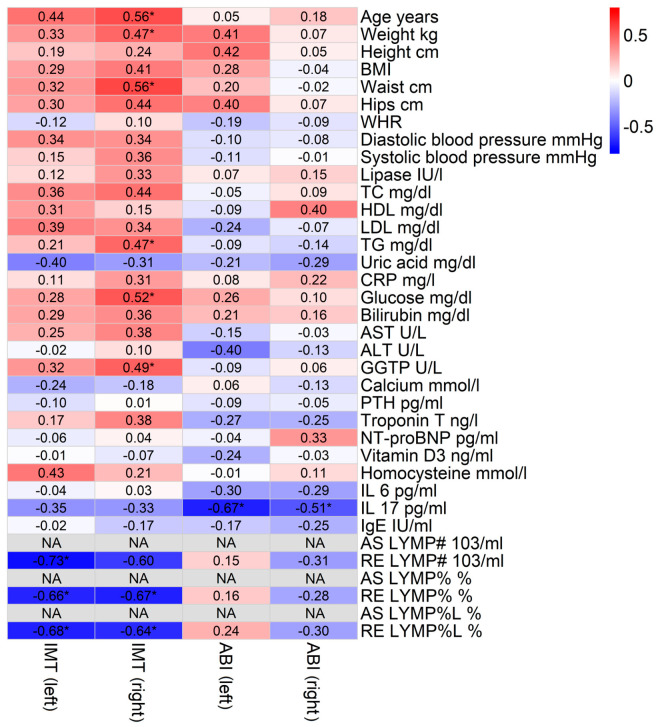
A heatmap illustrating the correlations between the mean values of IMT and ABI and demographic and clinical variables in the study group. Spearman’s rank correlation coefficients (Rho) are shown. Red indicates positive correlations and blue indicates negative correlations; the intensity of the color reflects the strength of the relationship. An asterisk (*) denotes statistically significant correlations (*p* < 0.05). Abbreviations: ABI—Ankle-Brachial Index; ALT—Alanine Aminotransferase; AS-LYMP#—absolute antibody-synthesizing lymphocyte count; AS-LYMP%—percentage of antibody-synthesizing lymphocytes; AS-LYMP%L—antibody-synthesizing lymphocytes as a percentage of lymphocytes; AST—Aspartate Aminotransferase; BMI—Body Mass Index; CRP—C-Reactive Protein; GGTP—Gamma-Glutamyl Transpeptidase; HDL—High-Density Lipoprotein Cholesterol; IgE—Immunoglobulin E; IL-6—Interleukin 6; IL-17—Interleukin 17; IMT—Intima-Media Thickness; LDL—Low-Density Lipoprotein Cholesterol; NT-proBNP—N-terminal pro-B-type Natriuretic Peptide; PTH—Parathyroid Hormone; RE-LYMP#—absolute reactive lymphocyte count; RE-LYMP%—percentage of reactive lymphocytes; RE-LYMP%L—reactive lymphocytes as a percentage of lymphocytes; TC—Total Cholesterol; TG—Triglycerides; WHR—Waist–Hip Ratio.

The mean IMT (right) showed significant positive correlations with age (R = 0.56; *p* < 0.05), weight (R = 0.47; *p* < 0.05), waist circumference (R = 0.56; *p* < 0.05), triglycerides (R = 0.47; *p* < 0.05), and glucose concentration (R = 0.52; *p* < 0.05). Significant negative correlations were observed between IMT (right) and AS-LYMP%L_(R = −0.67; *p* < 0.05) as well as RE-LYMP%L_(R = −0.64; *p* < 0.05). Additionally, IMT (left) was negatively correlated with AS-LYMP%L_(R = −0.66; *p* < 0.05) and RE-LYMP%L_(R = −0.68; *p* < 0.05). IL-17A showed significant negative correlations with ABI left (R = −0.672; *p* < 0.05) and ABI right (R = −0.512; *p* < 0.05) (Figure 2).

### 3.4. Venous Potency

A bilateral venous assessment (from the iliac veins to the ankles) showed no signs of new thrombosis. All examined deep veins were unobstructed, elastic, and free of residual thrombi in the lumen. Flow in femoral and popliteal veins in the standing position was spontaneous, showed normal respiratory phasing, and revealed no signs of reflux. Evaluation of the superficial veins, including the saphenous veins bilaterally, showed normal function with no abnormalities detected. No clinically relevant venous abnormalities were detected in either group, suggesting normal venous function in all participants. Only one patient in the study group had an atherosclerotic plaque, which decreased over time (after 3 months) and did not cause significant arterial occlusion.

### 3.5. Additional Analyses

**Figure 3:**
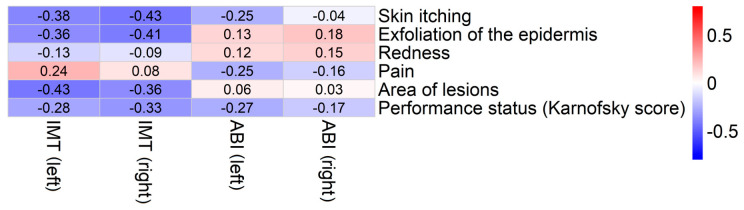
A heat map illustrating the relationships between the mean values of IMT and ABI and the clinical symptoms and functional status of patients in the study group. The values of Spearman’s rank correlation coefficients (Rho) are presented. Red indicates positive correlations, whilst blue indicates negative correlations; the intensity of the color corresponds to the strength of the relationship. Abbreviations: ABI—Ankle-Brachial Index; IMT—Intima-Media Thickness.

In this study, no significant correlations were found between assessed symptoms and IMT or ABI scores on either body side, as shown in Figure 3.

**Figure 4:**
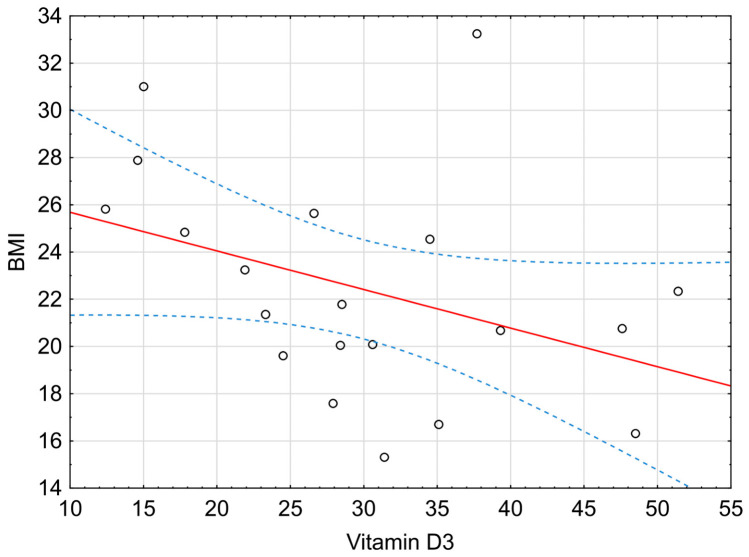
A scatter plot showing the statistically significant relationship between vitamin D3 concentration and BMI in the study group. The red line represents the linear regression line, whilst the blue dotted lines indicate the 95% confidence intervals. Each data point corresponds to an individual study participant.

Analysis of vitamin D3 levels revealed a significant negative correlation with body mass index (BMI) in the study group (R = −0.44; *p* = 0.0440), indicating that a higher BMI was associated with lower vitamin D3 levels (Table 5 and Figure 4).

## 4. Discussion

Our study demonstrated significantly lower LDL cholesterol levels in patients with inherited EDD, along with elevated IL-17A levels. This aligns with the literature data highlighting lipid alterations and inflammatory responses in various skin disorders [18,19,20]. The absence of significant differences in other assessed metabolic parameters in our study, including glucose and CRP levels, indicates that lipid profile variations are not merely reflections of systemic metabolic dysregulation. The observed reduced LDL cholesterol levels in patients with keratinization disorders contrast with findings from psoriasis, where dyslipidemia, particularly increased LDL cholesterol levels, has been frequently documented [21,22,23]. This suggests that while lipid disorders are prevalent in some inflammatory skin diseases, they may not be universally present across all conditions.

The finding of lower—rather than higher—LDL cholesterol in our cohort may initially appear to conflict with the notion of vascular vulnerability. However, the classical cholesterol-centric model of atherosclerosis may not be directly transferable to this population. Several inherited keratinization disorders are characterized by primary defects in epidermal and systemic lipid handling, which may reduce circulating LDL through mechanisms unrelated to cardiovascular protection. Moreover, standard lipid profiling captures only conventional fractions and may not reflect broader lipidomic alterations of potential cardiometabolic relevance. Accordingly, lower LDL cholesterol in EDD should not be interpreted as evidence of reduced vascular risk, but rather as a marker of disease-specific lipid metabolism that warrants further characterization.

Most forms of ichthyosis result from defects in the biosynthesis or processing of epidermal lipids and structural proteins essential for skin barrier integrity [24]. Mutations affecting enzymes involved in epidermal lipid metabolism, including ALOX12B, ALOXE3, CYP4F22, PNPLA1, ABCA12, CERS3, and SDR9C7, may contribute not only to barrier dysfunction and hyperkeratosis but also to broader disturbances in lipid handling [25,26]. Although direct evidence linking these pathways to systemic lipid abnormalities remains limited, recent transcriptomic data suggest that common ichthyoses skin transcriptomes show differential expression of genes related to atherosclerosis-linked products and lipid metabolism [27]. The lack of stronger associations between plasma lipid parameters and EDD may also be related to the small sample size, the substantial proportion of pediatric participants, and the low representation of inflammatory ichthyosis phenotypes in the study group. In addition, standard lipid profiling captures only conventional lipid fractions and may not reflect broader alterations in the lipidome that could be relevant to cardiometabolic risk.

Naxos syndrome (OMIM #601214) is an autosomal recessive cardiocutaneous disorder caused by mutations in *JUP*, encoding plakoglobin, and is defined by a triad of diffuse palmoplantar keratoderma, woolly hair, and arrhythmogenic right ventricular cardiomyopathy [28,29]. Woolly hair is present from birth and palmoplantar keratoderma appears within the first year of life, whereas cardiomyopathy usually manifests in adolescence but shows complete penetrance [28]. Cardiac involvement may be life-threatening: syncope and sustained ventricular tachycardia are common initial manifestations, sudden cardiac death may be the first presentation, and roughly one third of patients develop symptoms before the age of thirty [28]. This severe and progressive cardiac phenotype provides a clinical rationale for vascular assessment in affected patients. In our patient with Naxos syndrome, the presence of arrhythmogenic cardiomyopathy and severe cardiac complications, including heart transplantation, justified monitoring of vascular parameters such as carotid intima-media thickness. Although we did not identify structural vascular abnormalities, assessment of vascular health may be relevant in such patients as part of a broader cardiovascular surveillance strategy.

Administration of retinoids, particularly systemic, such as acitretin, is a common therapeutic approach to reduce scaling and hyperkeratosis. However, retinoids are known to significantly impact lipid metabolism, which can lead to elevated serum lipids, particularly triglyceride and total cholesterol levels [30]. For this reason, routine lipid monitoring is recommended for patients on long-term retinoid therapy. Generally, the study group did not receive any treatment for the 2 months preceding the study, except for Eucerin ointment with water used for generalized ichthyosis or 5% cholesterol urea ointment used for keratoses.

The subgroup analysis revealed that patients with generalized EDD exhibited significantly elevated IL-17A levels, in contrast to those with localized lesions. This may indicate a more systemic inflammatory state associated with generalized disease. It is widely known that elevated IL-17A levels may contribute to the chronic inflammatory processes observed in ichthyosis [31].

Barrier disruption in ichthyosis may also promote systemic immune activation. Loss of epidermal integrity induces the release of proinflammatory cytokines, including IL-1β, IL-6, and IL-23, which may stimulate IL-17 production by innate-like lymphocytes [32]. This may partly explain the elevated IL-17A levels observed in our cohort. Previous studies have shown upregulation of the Th17/Th22 axis in several forms of ichthyosis, with IL-17-related gene expression correlating with transepidermal water loss, erythema, and disease severity, suggesting that inflammatory activation in these disorders may extend beyond the skin [11,31,33,34,35].

Traditional cardiovascular risk factors, such as hypertension and hypercholesterolemia, have long been recognized as major contributors to coronary artery disease. However, increasing evidence indicates that inflammation is also a key component of atherogenesis and cardiovascular risk [36,37]. Elevated circulating IL-6 and high-sensitivity C-reactive protein have been associated with a higher incidence of atherosclerotic cardiovascular events [38,39]. More recently, the concept of inflammation-associated coronary artery disease has been proposed to describe patients in whom inflammatory disorders and/or inflammatory biomarkers may contribute to cardiovascular risk beyond traditional factors [40].

According to the European Society of Hypertension recommendations, IMT values exceeding 0.9 mm are indicative of early organ damage, while a normal range generally falls between 0.6 and 0.8 mm. Numerous studies highlighted the correlation between higher IMT and the severity of atherosclerosis [41,42,43]. We did not observe increased IMT in the study group compared to the control group, though. Nevertheless, correlations between carotid IMT and various metabolic parameters emphasize the complex, multifactorial nature of cardiovascular risk factors in this patient population. Specifically, the positive correlation of mean IMT with age, weight, and glucose levels may reflect established associations between metabolic syndrome components and atherosclerosis risk [44,45]. The absence of increased IMT despite elevated IL-17A concentrations may indicate an uncoupling between systemic inflammation and structural vascular remodeling in these rare disorders. Several explanations are plausible. First, the degree and duration of inflammation may not have reached the threshold required to induce measurable intima-media thickening. Second, our cohort was relatively young and included pediatric participants, in whom subclinical atherosclerosis is rarely detectable. Third, IMT reflects structural rather than functional vascular status and may be insensitive to early endothelial changes. Patients with Netherton syndrome, ichthyosis with confetti, and Naxos syndrome represent clinically important examples in which chronic inflammation, barrier dysfunction, and extracutaneous manifestations may influence vascular biology through mechanisms that differ from the traditional cholesterol-driven model of atherosclerosis. The negative correlation observed between ABI and IL-17A may suggest a potential functional association requiring further validation rather than an established structural disease.

Our findings also suggest intriguing interactions between inflammation and vascular health, particularly the significant negative correlation between ABI and IL-17 levels. This correlation may imply that higher levels of IL-17 could be detrimental to endothelial function and contribute to vascular abnormalities, a concept supported by studies linking IL-17 to endothelial dysfunction [46,47,48]. For example, Karbach et al. [47] observed the development of a severe psoriasis-like skin inflammation in mice with conditional overexpression of IL-17A in keratinocytes (K14-IL-17A(ind/+)), accompanied by elevated reactive oxygen species and increased levels of circulating CD11b(+) inflammatory leukocytes. These mice also exhibited endothelial dysfunction, elevated systolic blood pressure, left ventricular hypertrophy, and a shorter lifespan compared to controls. Anyway, this correlation may suggest potential associations requiring further validation rather than established causal relationships.

Our study found no evidence of new thrombotic formations within the venous system. All examined deep veins were clear, flexible, and free of residual thrombi. The superficial veins also demonstrated normal function, with no detected abnormalities. However, the existing literature suggests that psoriasis is associated with an increased risk of venous thromboembolism (VTE) and other thrombotic events [49,50,51]. Confounder-adjusted data indicate a higher prevalence of VTE among individuals with psoriasis, though this association varies depending on the number of adjusted confounders and the severity of psoriasis [49]. A meta-analysis of five studies reported a pooled risk ratio of 1.29 (95% CI: 0.92–1.81) for VTE in patients with psoriasis, although the analysis showed high statistical heterogeneity (I^2^ = 97%) [49].

Despite the limited literature on cardiovascular assessments in patients with selected inherited EDD, our study attempted to address this gap by providing insights into potential cardiovascular risks in this population. According to the 2018 guidelines of the American Heart Association and the American College of Cardiology, psoriasis and related conditions are classified as “cardiovascular risk amplifiers” [52]. The new European guidelines also stress the role of chronic inflammatory diseases in the pathogenesis of atherosclerotic CVD [53]. Given the high mortality associated with CVD worldwide, routine vascular evaluations are essential for patients at elevated risk to detect early or preclinical cardiovascular changes.

The lack of significance in terms of IMT stems from the age of both groups and, consequently, the less frequently observed classical environmental cardiovascular risk factors. This suggests that chronic inflammation typical of selected EDD is an insufficient factor to indicate subclinical atherosclerosis if not accompanied by the other cardiovascular risk factors.

Similar conclusions can be drawn by analyzing the results of Doppler ultrasonography of the lower extremity veins, which did not demonstrate VTE in our study. It is likely that this is due to the absence of concomitant diseases predisposing patients to VTE and occasional exposure to reversible risk factors for VTE (e.g., immobilization, bone fractures) in both patient groups.

The inverse association between vitamin D3 levels and BMI observed in our cohort may reflect broader links between vitamin D deficiency and metabolic risk. Vitamin D deficiency has been associated with obesity and several cardiovascular risk pathways, including alterations in adipose tissue metabolism, activation of the renin–angiotensin–aldosterone system, secondary hyperparathyroidism, and promotion of atherogenesis, although a definitive causal relationship with cardiovascular disease has not been established [54,55,56]. In our study, however, vitamin D3 was not associated with IMT or ABI, suggesting that its potential relevance may be indirect and related more to metabolic status than to detectable vascular alterations at this stage.

This study has several limitations that should be considered. The relatively small sample size may limit the generalizability of our findings. However, the inclusion of pediatric patients adds valuable insight. Additionally, the heterogeneity of the study group, which reflects the rarity of EDD, may introduce variability that affects the interpretation of IMT score associations. Also, patients receiving systemic agents that affect lipid or inflammatory parameters were excluded and no participant received systemic retinoids (e.g., acitretin) during or within two months before assessment; residual effects of prior topical therapy cannot be fully excluded. Furthermore, IMT and short-term IMT/ABI re-assessment are insensitive to early functional vascular change; endothelial function testing and longer follow-up would be more informative in future work. Also, given the exploratory nature of the study and the number of comparisons performed, the absence of adjustment for multiple testing increases the risk of type I error; therefore, the findings should be interpreted as hypothesis-generating.

## 5. Conclusions

In patients with selected inherited EDD, we identified a distinct biochemical signature characterized by lower LDL cholesterol and higher IL-17A concentrations, in the absence of structural arterial abnormalities (comparable carotid IMT and ABI, with no short-term change). Rather than indicating established atherosclerosis, these findings point to disease-specific alterations of lipid and inflammatory homeostasis whose long-term vascular implications remain uncertain. Future studies should incorporate endothelial function testing and longer-term follow-up, rather than relying on IMT alone, and should give particular attention to syndromic forms with known extracutaneous involvement, such as Naxos syndrome, within a broader cardiovascular surveillance framework.

## Figures and Tables

**Table 1:** Baseline characteristics of the study group regarding symptoms and performance status.

Variables	Study Group [*n* = 20]
Occurrence of the lesions on exposed parts of the body	
No	11 (55%)
Yes	9 (45%)
Location of scales	
Whole body	8 (40%)
Whole body (except face, hands and feet)	1 (5%)
Hands and feet	2 (10%)
Hands, feet, spine	1 (5%)
Hands, feet, Achilles tendon	1 (5%)
Elbows, knees, buttocks	1 (5%)
Heels and hands	1 (5%)
Hands, legs	1 (5%)
Scalp, body	1 (5%)
Feet	2 (10%)
Feet, ankles, calves, knees	1 (5%)
Infiltration	
No	5 (25%)
Yes	15 (75%)
Itching	5.0 [3.2–7.8]
	(0.0–10.0)
Exfoliation	6.5 [3.0–8.0]
	(0.0–10.0)
Redness	4.0 [3.0–5.8]
	(0.0–10.0)
Pain	2.0 [0.0–5.0]
	(0.0–8.0)
Lesion surface	35.0 [5.9–80.0]
	(1.0–100.0)
Lesion extent	
Limited	8 (40%)
Generalized	12 (60%)
Performance status	90.0 [71.7–90.0]
	(6.0–100.0)

Values are presented as Median [IQR] (min. − max.) or *n* (%).

**Table 2:** Baseline characteristics and comparison of the control and study groups (including sub-groups) in terms of demographic, cardiovascular risk factors, clinical, and laboratory variables.

Variable	Study Group [*n* = 20]	Control (C) [*n* = 20]	*p*	Localized Lesions (L) [*n* = 8]	Generalized Lesions (G) [*n* = 12]	*p* (G vs. L)	*p* (G vs. C)	*p* (L vs. C)
Sex			1.0000			0.7206	0.9847	0.9801
Female	9 (45)	9 (45)		4 (50)	5 (41.7)			
Male	11 (55)	11 (55)		4 (50)	7 (58.3)			
Smoking			0.7373			0.4500	0.6527	0.6419
Never	16 (80)	14 (70)		6 (75)	10 (83.3)			
Active	3 (15)	4 (20)		2 (25)	1 (8.3)			
Former	1 (5)	2 (10)		0 (0)	1 (8.3)			
Pack-years (N/a *n* = 30)	10 [5.2–15] (0.3–20)	5 [4.5–20] (2.5–20)	0.8281	15 [10–20] (10–20)	5.2 [0.3–10] (0.3–10)	0.2207	0.4998	0.4025
Diet			0.7470			0.7208	0.6112	0.9162
Poor	7 (35)	5 (25)		2 (25)	5 (41.7)			
Average	7 (35)	9 (45)		3 (27.5)	4 (33.3)			
Good	6 (30)	6 (30)		3 (37.5)	3 (25)			
Physical activity			0.3346			0.2325	0.6139	0.1141
No	5 (25)	3 (15)		1 (12.5)	4 (33.3)			
Low	4 (20)	1 (5)		3 (37.5)	1 (8.3)			
Medium	9 (45)	12 (60)		4 (50)	5 (41.7)			
High	2 (10)	4 (20)		0 (0)	2 (16.7)			
CVD			0.4801			0.6424	0.2399	1.0000
Negative	13 (65)	16 (80)		6 (75)	7 (58.3)			
Positive	7 (35)	4 (20)		2 (25)	5 (41.7)			
Age [years]	25.5 [14–40.5] (2.5–49)	25.5 [14.5–40] (2.5–50)	0.9680	27 [14–38] (10–47)	25.5 [14.5–41.5] (2.5–49)	0.9692	0.6044	0.5327
Weight [kg]	63 [52–73] (14.5–105)	62.5 [54–72.5] (15–104)	0.9893	68 [58.5–75.5] (30–103)	61.5 [39.4–70.5] (14.5–105)	0.3542	0.6591	0.7842
Height [cm]	169.5 [155.5–177] (86–184)	170.5 [157–177.5] (86–186)	0.8831	174 [161–176] (140–180)	168 [145.5–179] (86–184)	0.6431	0.6044	0.6005
BMI	21.6 [19.8–25.2] (15.3–33.3)	21.9 [20.4–25] (13.7–33.2)	0.9467	23.9 [18.8–26.8] (15.3–33.3)	21.1 [19.8–23.6] (16.3–31)	0.5371	0.8631	0.5327
Waist [cm]	84 [73–93.5] (55–111)	81 [74–93] (51–110)	0.8201	89 [73–99.5] (60–108)	80.5 [72–90] (55–111)	0.4870	0.4772	0.8227
Hips [cm]	98 [86.5–105] (52–115)	101 [91–105] (51–114)	0.7180	101.5 [88.5–108.5] (72–115)	95 [81–104] (52–114)	0.4174	0.2391	0.6718
WHR	0.9 [0.8–1] (0.8–1.1)	0.9 [0.8–0.9] (0.7–1)	0.2888	0.9 [0.8–0.9] (0.8–1)	0.9 [0.8–1] (0.8–1.1)	0.4858	0.5015	0.9801
Systolic blood pressure [mmHg]	117.5 [104.5–131] (86–160)	117 [110–120.5] (80–127)	0.6588	114.5 [104.5–128] (86–152)	117.5 [102.5–131] (90–160)	0.8467	0.6044	0.5327
Diastolic blood pressure [mmHg]	72.5 [61–82.5] (54–110)	77.5 [70–84] (55–91)	0.4777	70 [59–85] (54–98)	72.5 [63.5–82.5] (60–110)	0.5614	0.3867	0.8617
Lipase [IU/L]	26 [18–38.1] (13–141)	26 [23–36.5] (16–46)	0.6205	27 [19–45] (14–58)	24.5 [18–35.6] (13–141)	0.6431	0.1070	0.1653
TC [mg/dL]	146.2 [120.5–185.7] (98.5–248.8)	178.7 [157.6–184.9] (94.7–211.3)	0.0596	134.5 [117–190.8] (99.7–248.8)	151.2 [122.7–185.7] (98.5–219.9)	0.8170	0.5015	0.3542
HDL [mg/dL]	51 [45.3–65.1] (17.4–81.2)	50.8 [45.1–66.5] (32–81)	0.9893	57.4 [49.7–70.2] (42.5–81.2)	50.8 [40.3–60.3] (17.4–72)	0.1897	0.0656	0.0887
LDL [mg/dL]	75 [59–100] (43–144)	105.5 [92.3–113.5] (57–152)	0.0262 *	71.5 [58–105] (43–144)	85 [62.5–99] (52–133)	0.6494	0.6872	0.4385
TG [mg/dL]	68 [54–134.5] (36–542)	86 [78–110.5] (37–150)	0.4777	68 [44.5–127] (36–167)	67 [59–167.5] (44–542)	0.5369	0.9847	0.9801
Glucose [mg/dL]	87.1 [85.2–91] (78.1–100)	84.6 [81.1–88] (74–99.9)	0.0524	90.4 [87.6–94.1] (85–95.9)	85.9 [84.2–88.8] (78.1–100)	0.0537	0.3867	0.0114 *
Bilirubin [mg/dL]	0.6 [0.4–0.9] (0.3–1.6)	0.6 [0.4–0.7] (0.2–1.1)	0.5315	0.7 [0.5–1.5] (0.4–1.6)	0.5 [0.4–0.7] (0.3–1.1)	0.1225	0.8574	0.1469
AST [U/L]	17.9 [14.1–25.7] (10.2–55.8)	20.8 [18.3–25.7] (11.3–41.4)	0.1738	18.3 [15.6–21.1] (13–29)	16.9 [12.9–29.8] (10.2–55.8)	0.8170	0.3073	0.2177
ALT [U/L]	12.8 [10.2–20.9] (7–49.3)	18 [12.3–23.1] (8–30)	0.1937	10.9 [10.2–18.3] (9.2–27)	14 [9.7–23.3] (7–49.3)	0.5371	0.5353	0.1063
GGTP [U/L]	16.5 [12.5–46.5] (8–124)	19 [11–30] (6.9–70)	0.9254	18 [13.5–30] (12–75)	16 [10–62.5] (8–124)	0.5621	0.8933	0.7086
Troponin T [ng/L]	3.8 [3–4.9] (3–6.3)	4.4 [3–5.6] (3–18.3)	0.3547	4.2 [3–4.7] (3–5.1)	3.6 [3–5.8] (3–6.3)	0.8757	0.5015	0.4092
NT-proBNP [pg/mL]	57 [14–111] (10–1496)	45.5 [21–75] (10–114)	0.6205	54.5 [12–92] (10–185)	57 [14.5–131.5] (11–1496)	0.6158	0.5518	0.9009
Homocysteine [μmol/L]	9.8 [7.2–13.2] (6.1–18.2)	9.5 [6.7–12.2] (4.6–16)	0.4777	10.1 [7.5–12.5] (6.7–15.4)	9.8 [7.2–13.2] (6.1–18.2)	0.8774	0.5778	0.5661
Uric acid [mg/dL]	5.5 [4.9–6.4] (2.6–9)	2.3 [2.2–2.4] (2.2–2.5)	0.8201	5.5 [4.7–6.3] (3.8–9)	5.6 [5.2–6.9] (2.6–8.1)	0.5883	0.7445	1.000
Calcium [mmol/L]	2.3 [2.3–2.4] (2.2–4)	2.3 [2.2–2.4] (2.2–2.5)	0.3408	2.4 [2.3–2.4] (2.2–2.5)	2.4 [2.3–2.5] (2.2–4)	0.5366	0.6872	0.2177
PTH [pg/mL]	32.7 [24.2–43.8] (13.4–56.1)	28.5 [24.8–35.1] (15.2–67)	0.6588	26.8 [24.2–38.5] (13.4–52.5)	39.3 [23.6–44.7] (16–56.1)	0.3961	0.4083	0.8227
Vitamin D3 [ng/mL]	28.5 [22.6–36.4] (12.4–51.4)	28.2 [22.7–34.2] (16.3–61.8)	0.9467	28.2 [24.3–32.9] (14.6–37.7)	29.6 [20.6–43.5] (12.4–51.4)	0.5892	0.9542	0.8227
CRP [mg/L]	1 [0.6–2.3] (0.5–6)	1.4 [0.7–1.8] (0.4–7.9)	0.8620	1 [0.6–3.5] (0.6–6)	1 [0.7–2.3] (0.5–3.4)	0.8755	0.8933	0.9009
IL-6 [pg/mL]	2 [1.5–2.7] (1.5–16.7)	1.5 [1.5–2.4] (1.5–5.8)	0.0965	1.6 [1.5–2.4] (1.5–3)	2.1 [1.7–3.1] (1.5–16.7)	0.2912	0.0699	0.4385
IL-17A [pg/mL]	0.1 [0.1–0.1] (0.1–0.4)	0.2 [0.1–0.5] (0.1–7.9)	0.0047 *	0.1 [0.1–0.2] (0.1–0.3)	0.3 [0.1–0.8] (0.1–7.9)	0.0068 *	0.0002 *	0.5661

Values are presented as median [IQR] (min–max) or *n* (%); *—statistically significant result. Abbreviations: AST—Aspartate Aminotransferase; ALT—Alanine Aminotransferase; BMI—Body Mass Index; CRP—C-Reactive Protein; CVD—Cardiovascular Disease; GGTP— Gamma-glutamyl transpeptidase; HDL—High-Density Lipoprotein Cholesterol; IL-6—Interleukin-6; IL-17A—Interleukin-17A; LDL—Low-Density Lipoprotein Cholesterol; NT-proBNP—N-terminal pro–B-type Natriuretic Peptide; PTH—Parathyroid Hormone; TC—Total Cholesterol; TG—Triglycerides; WHR—Waist–Hip Ratio.

**Table 3:** Comparison of intima-media thickness and ankle-brachial index between the study groups and subgroups.

Variable	Control (C) [*n* = 20]	Study Group (S) [*n* = 20]	Generalized Disease (G) [*n* = 12]	Limited Disease (L) [*n* = 8]	*p* (C vs. S)	*p* (C vs. G)	*p* (C vs. L)	*p* (G vs. L)
IMT (left)	0.5 [0.4–0.6] (0.3–0.8)	0.5 [0.4–0.6] (0.3–1)	0.5 [0.3–0.5] (0.3–0.6)	0.6 [0.4–0.6] (0.3–1)	0.5662	0.2385	0.7204	0.0813
IMT (right)	0.5 [0.4–0.6] (0.4–0.7)	0.5 [0.4–0.6] (0.3–0.7)	0.4 [0.4–0.5] (0.3–0.6)	0.6 [0.5–0.6] (0.4–0.7)	0.9529	0.4499	0.3286	0.1239
ABI (left)	1 [1–1.1] (1–1.2)	1.1 [1–1.1] (1–1.2)	1.1 [1–1.1] (1–1.2)	1.1 [1–1.1] (1–1.2)	0.4330	0.8809	0.2490	0.3302
ABI (right)	1.1 [1–1.1] (1–1.1)	1.1 [1–1.1] (0.9–1.4)	1.1 [1–1.1] (1–1.2)	1.1 [1.1–1.1] (0.9–1.2)	0.0871	0.2831	0.0863	0.5606

Values are presented as median [IQR] (min–max). Abbreviations: ABI—Ankle-Brachial Index; IMT—Intima-Media Thickness.

**Table 4:** Comparison in terms of means of the results of measurements of the intima-media complex and ankle-brachial index in the study group at two subsequent time points.

Variable	First Measurement [*n* = 20]	Second Measurement [*n* = 20]	*p*
IMT (left)	0.5 [0.3–0.9] (0.3–1.0)	0.5 [0.4–0.9] (0.4–1.0)	0.3750
IMT (right)	0.5 [0.4–0.7] (0.4–0.7)	0.5 [0.4–0.7] (0.4–0.6)	1.0000
ABI (left)	1.1 [1–1.2] (1–1.2)	1.1 [1–1.1] (1–1.1)	0.3125
ABI (right)	1.1 [1–1.2] (0.9–1.2)	1.1 [1–1.1] (1–1.1)	0.3125

Values are presented as median [IQR] (min–max). Abbreviations: ABI—Ankle-Brachial Index; IMT—Intima-Media Thickness.

**Table 5:** Correlations between Vitamin D3 concentration and demographic and clinical variables in the study group.

Variable	Study Group [*n* = 20]	
	Vitamin D3	
	Rho	*p*
Age [years]	−0.19	0.4036
Weight [kg]	−0.21	0.3668
Height [cm]	0.08	0.7302
BMI	−0.44	0.0440
Waist [cm]	−0.17	0.4517
Hips [cm]	−0.28	0.2132
WHR	0.21	0.3722
Diastolic blood pressure [mmHg]	−0.35	0.1228
Systolic blood pressure [mmHg]	−0.26	0.2464
Lipase [IU/L]	0.07	0.7602
TC [mg/dL]	−0.22	0.3391
HDL [mg/dL]	0.11	0.6218
LDL [mg/dL]	−0.17	0.4695
TG [mg/dL]	−0.26	0.2541
Glucose [mg/dL]	−0.14	0.5574
Bilirubin [mg/dL]	−0.19	0.4177
AST [U/L]	0.05	0.8453
ALT [U/L]	0.21	0.3695
GGTP [U/L]	0.04	0.8598
Troponin T [ng/L]	0.00	0.9909
NT-proBNP [pg/mL]	−0.12	0.5899
Homocysteine [umol/L]	−0.02	0.9154
Uric acid [mg/dL]	0.03	0.9042
Calcium [mmol/L]	0.24	0.2897
PTH [pg/mL]	−0.13	0.5632
CRP [mg/L]	−0.13	0.5795
IL-6 [pg/mL]	−0.19	0.4215
IL-17 [pg/mL]	0.22	0.3427
IgE [IU/mL]	−0.08	0.7285
AS-LYMP# [10^3^/uL]	n/a	n/a
RE-LYMP# [10^3^/uL]	−0.25	0.4846
AS-LYMP% [%]	n/a	n/a
RE-LYMP% [%]	−0.18	0.6250
IMT (left)	0.03	0.9058
IMT (right)	−0.05	0.8263
ABI (left)	−0.23	0.3593
ABI (right)	−0.03	0.9055

Abbreviations: ABI—Ankle-Brachial Index; ALT—Alanine Aminotransferase; AS-LYMP#—absolute antibody-synthesizing lymphocyte count; AS-LYMP%—percentage of antibody-synthesizing lymphocytes; AST—Aspartate Aminotransferase; BMI—Body Mass Index; CRP—C-Reactive Protein; GGTP—Gamma-Glutamyl Transpeptidase; HDL—High-Density Lipoprotein Cholesterol; IgE—Immunoglobulin E; IL-6—Interleukin 6; IL-17—Interleukin 17; IMT—Intima-Media Thickness; LDL—Low-Density Lipoprotein Cholesterol; NT-proBNP—N-terminal pro-B-type Natriuretic Peptide; PTH—Parathyroid Hormone; RE-LYMP#—absolute reactive lymphocyte count; RE-LYMP%—percentage of reactive lymphocytes; TC—Total Cholesterol; TG—Triglycerides; WHR—Waist–Hip Ratio.

## Data Availability

The data presented in this study are available from the corresponding author upon reasonable request.
